# Genomic epidemiology analysis of extremely drug-resistant tuberculosis in Shanghai, China

**DOI:** 10.1080/22221751.2025.2521842

**Published:** 2025-06-23

**Authors:** Xiaoyu Lu, Yuan Jiang, Yanping Liu, Junhong Chen, Yinglin Lao, Jing Li, Yangyi Zhang, Nan Li, Lili Wang, Chenlei Yu, Qi Ye, Wei Wei, Jiale Deng, Xin Shen, Chongguang Yang

**Affiliations:** a School of Public Health (Shenzhen), Shenzhen Key Laboratory of Pathogenic Microbes and Biosafety, Shenzhen Campus of Sun Yat-sen University, Sun Yat-sen University, Guangdong, People’s Republic of China; b Division of TB and HIV/AIDS Prevention, Shanghai Municipal Center for Disease Control and Prevention, Shanghai, People’s Republic of China

**Keywords:** EDR-TB, transmission, whole-genome sequencing, compensatory mutations, China

## Abstract

Tuberculosis (TB), particularly extremely drug-resistant TB (EDR-TB), remains a significant public health concern worldwide. Understanding the transmission patterns and epidemiological characteristics of EDR-TB is vital for effective disease control. Between 1 January 2006 and 31 December 2018, we collected clinical *M. tuberculosis* strains in Shanghai, with whole-genome sequencing performed on 58 identified clinical EDR-TB strains. We analyzed EDR-related genetic mutations, conducted phylogenetic analyses, and examined bacterial and epidemiological factors that influence their transmission. Among these 58 EDR patients, 43.1% (25/58) were aged 45–64 years, with a median age of 51 years (interquartile range, IQR, 29–59 years). About two-thirds of the EDR-TB patients were residents. We observed a clustering rate of 44.8% (26/58) among EDR strains. Logistic regression analysis indicated a higher risk of recent EDR-TB transmission among the strains with the drug-resistant compensatory mutations. The primary mode of EDR-TB transmission in the study setting was recent, direct person-to-person spread of drug-resistant strains, as evidenced by high clustering rates and the presence of identical resistance mutations among clustered cases.

Drug-resistant tuberculosis (TB) is a major contributor to antimicrobial resistance globally and remains a public health threat, with approximately 500,000 new cases annually [1]. Recently, the global spread of highly drug-resistant strains has garnered significant attention [2]. Extremely drug-resistant TB (EDR-TB), one of the most severe form of drug resistance, has been reported worldwide and is defined as multidrug-resistant TB (MDR-TB) with additional resistance to any fluoroquinolone and any of three second-line injectable drugs [3]. This high level of resistance severely limits treatment options, necessitating complex, toxic, and expensive regimens [4]. Treatment success rates for most affected populations remain below 40%, and mortality rates range from 50% to 80% [5,6]. EDR-TB poses a severe public health challenge due to its complex treatment regimens, which place a significant burden on global healthcare systems [7,8]. While not more contagious than drug-susceptible TB theoretically, recent studies highlight an increased risk of its transmission in community settings [1,9]. This study used epidemiological and whole-genome sequencing (WGS) data with the aim of investigating the transmission patterns and the risk factors of patients with EDR-TB in Shanghai, China.

From 1 January 2006 to 31 December 2018, Shanghai recorded cases of EDR pulmonary TB in individuals aged 15 years and older, confirmed through culture methods. Sputum samples from all patients were collected prior to the commencement of treatment and dispatched to the Tuberculosis Reference Laboratory at the Shanghai Center for Disease Control and Prevention for drug susceptibility testing (DST) and isolates preservation. Furthermore, epidemiological data, along with demographic, clinical, and microbiological information, were obtained from the National TB surveillance system. Patient delay was defined as the period between the onset of symptoms and the initiation of medical consultation. The term resident referred to individuals registered with a household in Shanghai, whereas migrant denoted those without household registration in Shanghai who originated from other regions. Genomic DNA was extracted using the cetyltrimethylammonium bromide method. Paired-end DNA sequencing was performed on the Hiseq2500 platform (Illumina, San Diego, CA, USA) with an expected coverage of 100× or greater. WGS data were initially processed using FastQC (version 0.12.1) to perform quality control. The refined sequencing reads were then aligned to the reference genome H37Rv (NC_000962.3) using Bowtie2 (version 2.3.1). TB-Profiler (version 4.1.0) was used to detect genotypic drug-resistance mutations to anti-TB drugs. Previous studies have reported that isolates with ≤12 single-nucleotide polymorphisms are consistent with transmission. A phylogenetic tree was constructed using RAxML (version 1.0.2) under the maximum likelihood method with the GTR substitution model and 500 bootstrap replicates. To identify compensatory mutations, we used a catalogue of putative compensatory mutations based on previously published studies [10,11]. Only high-confidence compensatory mutations were considered, including compensatory mutations in *rpoA, rpoB, rpoC t*hat were acquired independently at least twice in a large-scale phylogenetic analysis.

Among the 58 enrolled patients with EDR-TB, the majority were men (86.2%), with a median age of 51 (IQR: 29–59) years, and13.8% of them were elderly patients with age of 65 years or older. More patients were from the resident population than from migrant groups (67.2% vs. 32.8%). Diagnostic delays exceeding four weeks occurred in 50.0% (29/58) of cases, and 58.6% of patients had positive sputum smears. Regarding treatment outcomes, 24.1% (14/58) experienced treatment failure or death, whereas 58.6% (34/58) had completed treatment or achieved recovery. Genomic analysis revealed that 91.4% (53/58) of the strains belonged to Lineage 2 (L2), predominantly L2.2(23/58) and L2.3 (30/58), whereas the remaining 8.6% (5/58) were classified as Lineage 4 (L4) (Figure 1(A)). We identified that 70.0% (40/58) of the EDR-TB strains harboured compensatory mutations, mainly in the *rpoB* (24/58) and *rpoC* (22/58) genes. Additionally, two strains exhibited compensatory mutations in the *rpoA* gene (Figure 1(A)). We observed a clustering rate of 44.8% (26/58) among the EDR strains, resulting in seven distinct clusters. The largest cluster (Cluster-7, Figure 1), comprising nine patients, consisted entirely of resident TB patients and primarily affected older individuals. The longest transmission span within these clusters was up to 12 years (Figure 1(D)). Almost all the clustered patients that did not reside on the same street; we observed a median distance of 19.1 km between their residences of patient pairs, suggesting casual contact that might lead to transmission (Figure 1(B)). In the Cluster-7, most patients (66.7%, 6/9) experienced diagnostic delays of more than four weeks, and 55.6% (5/9) were new cases (Figure 1(D)). Half of the patients in this cluster died within one year of diagnosis. All isolates in this large cluster belonged to sublineage 2.2. Finally, we used logistic regression to identify risk factors associated with genomic clustering. The results revealed significant associations between the presence of compensatory mutations (aOR 5.96, 95% CI 1.48–23.96, *P* = 0.01) (Figure 1(C)). Among the clustered EDR-TB patients, 61.5% (16/26) experienced diagnostic delays of more than four weeks, and 69.2% (18/26) had positive sputum cultures. In addition, 57.6% (15/26) of the clustered patients were treatment-naïve, and 70.6% (20/26) were from the resident population. One of the seven genomic clusters involved transmission between residents and migrants, including a five-patient cluster (Cluster-3) entirely composed of migrants from the same province (Supplementary Table 1). Regarding patient outcomes, the majority of clustered patients (13/26) were cured; however, treatment failed or death occurred in five patients (Figure 1(D)).

**← Figure 1.**
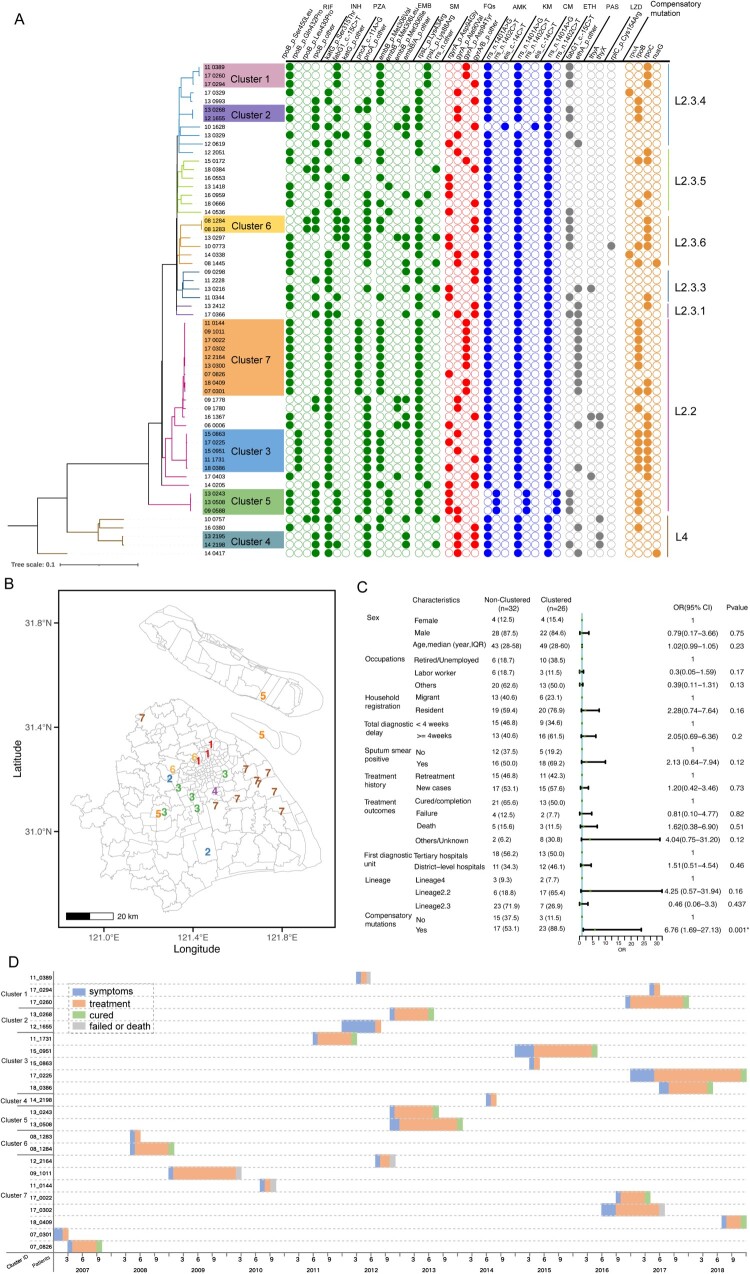
Epidemiological characteristics, phylogenetic tree and drug resistance profiles of EDR patients. (A) Phylogenetic Tree and Drug Resistance Profiles of EDR Patients. The phylogenetic tree illustrates the evolutionary relationships among EDR-TB strains, categorized into distinct lineages and represented by colored branches. Each colored bar corresponds to a lineage, with annotations indicating sublineages. The time scale bar at the top represents evolutionary distance. (B) Spatial distribution of clusters presented in map. There are two EDR patients with cluster id 4, and the other patient has missing address data. (C) Univariate Logistic Analysis of Risk Factors for EDR Transmission. Note: Five patients had missing data on diagnostic delay, seven had missing sputum culture results, and four lacked records on the First diagnostic unit. (D) The time (by year and month) of onset of symptoms, diagnosis, and treatment of the seven EDR-TB clusters. Diagnostic delay is indicated in blue, the treatment course in orange, treatment completion in green, and treatment failure or death in gray.

## Discussion

Our findings reveal that EDR-TB in this setting predominantly affects middle-aged and elderly resident men, many of whom experience significant diagnostic delays and remain infectious. Most strains belonged to Lineage 2 and carried compensatory mutations, which were strongly linked to transmission clustering [12]. The identification of several genomic clusters, including a large, long-standing one involving only resident patients with high mortality and delayed diagnosis, highlights ongoing community transmission. These findings underscore the need for earlier detection, enhanced case management, and the integration of genomic surveillance into EDR-TB control strategies.

Our findings indicate that casual contact may represent one of multiple pathways contributing to the transmission of EDR-TB in Shanghai. Meanwhile, the diagnostic delays were common among both resident and migrant patients, with nearly half of the patients waiting more than four weeks to receive a confirmed diagnosis. Although our findings did not reveal a significant impact of diagnostic delays on the spread of EDR-TB, such long delays not only increase the disease burden on patients but also facilitate the spread of drug-resistant strains [13]. Furthermore, those undiagnosed patients may unknowingly transmit the bacteria within their communities [14]. Rare mutations linked to bedaquiline and linezolid emphasize the continued reliance on fluoroquinolones and injectables, but rising resistance necessitates novel regimens, including new drugs, optimized combinations, and dose adjustments. Our study also observed that EDR-TB strains with compensatory mutations were more likely to be involved in transmission. These mutations, primarily affecting RNA polymerase subunits, can partially restore enzymatic function impaired by resistance mutations, reducing fitness costs and potentially enhancing transmissibility [15]. There are several limitations. Although a genomic clustering rate of 44.8% was observed for EDR-TB, many patients could not be captured by the current passive surveillance system or be diagnosed in other regions, which can lead to an underestimation of the clustering rate [16]. In addition, the small sample size could also limit the generalizability of the findings to a broader population. Overall, this study highlights the critical role of molecular surveillance in managing EDR-TB and contributes valuable insights for the development of novel diagnostic tools and therapeutic strategies. Continued research is crucial for further elucidating transmission mechanisms and informing the development of effective global control measures.

## Supplementary Material

Supplementary Table 1.xlsx

## Data Availability

The genomic data are available under accession number PRJCA040148 in the Genome Sequence Archive at the National Genomics Data Center, part of the China National Center for Bioinformation.
